# The Endocannabinoid System as a Pharmacological Target for New Cancer Therapies

**DOI:** 10.3390/cancers13225701

**Published:** 2021-11-15

**Authors:** Robert Ramer, Felix Wittig, Burkhard Hinz

**Affiliations:** Institute of Pharmacology and Toxicology, Rostock University Medical Centre, Schillingallee 70, 18057 Rostock, Germany; robert.ramer@med.uni-rostock.de (R.R.); felix.wittig@med.uni-rostock.de (F.W.)

**Keywords:** cannabinoids, endocannabinoid system, FAAH inhibitors, MAGL inhibitors, cancer, apoptosis, autophagy, tumour cell proliferation, tumour cell invasion, metastasis, angiogenesis, tumour-immune interactions

## Abstract

**Simple Summary:**

Cannabinoids have been shown to suppress tumour cell proliferation, tumour invasion, metastasis, angiogenesis, chemoresistance and epithelial-mesenchymal transition and to induce tumour cell apoptosis, autophagy and immune response. This review focuses on the current status of investigations on the impact of inhibitors of endocannabinoid-degrading enzymes on tumour growth and spread in preclinical oncology research.

**Abstract:**

Despite the long history of cannabinoid use for medicinal and ritual purposes, an endogenous system of cannabinoid-controlled receptors, as well as their ligands and the enzymes that synthesise and degrade them, was only discovered in the 1990s. Since then, the endocannabinoid system has attracted widespread scientific interest regarding new pharmacological targets in cancer treatment among other reasons. Meanwhile, extensive preclinical studies have shown that cannabinoids have an inhibitory effect on tumour cell proliferation, tumour invasion, metastasis, angiogenesis, chemoresistance and epithelial-mesenchymal transition (EMT) and induce tumour cell apoptosis and autophagy as well as immune response. Appropriate cannabinoid compounds could moreover be useful for cancer patients as potential combination partners with other chemotherapeutic agents to increase their efficacy while reducing unwanted side effects. In addition to the direct activation of cannabinoid receptors through the exogenous application of corresponding agonists, another strategy is to activate these receptors by increasing the endocannabinoid levels at the corresponding pathological hotspots. Indeed, a number of studies accordingly showed an inhibitory effect of blockers of the endocannabinoid-degrading enzymes fatty acid amide hydrolase (FAAH) and monoacylglycerol lipase (MAGL) on tumour development and spread. This review summarises the relevant preclinical studies with FAAH and MAGL inhibitors compared to studies with cannabinoids and provides an overview of the regulation of the endocannabinoid system in cancer.

## 1. Introduction

Individual components of the endocannabinoid system have been intensively studied in recent decades and evaluated as potential targets of pharmacological interventions in systemic tumour therapy. *N*-arachidonoylethanolamine (anandamide, AEA) and 2-arachidonoylglycerol (2-AG) were the first lipids discovered as endogenously synthesised agonists at cannabinoid receptors [1,2]. Other endogenously formed cannabinoid compounds are 2-arachidonoylglycerol ether (noladin ether, 2-AGE) [3], *N*-arachidonoyldopamine (NADA) [4] and O-arachidonoylethanolamine (virodhamine) [5]. Cannabinoid-triggered receptors include the pertussis toxin-sensitive, G_i/o_ protein-coupled cannabinoid receptors CB_1_ and CB_2_ [6,7]. The phytocannabinoid Δ^9^-tetrahydrocannabinol (THC), the main psychoactive constituent of *Cannabis sativa* L., has the properties of a partial agonist at the CB_1_ receptor and a full agonist at the CB_2_ receptor with K_i_ values in the nanomolar range, while the non-psychoactive phytocannabinoid cannabidiol (CBD) has a much weaker affinity for cannabinoid receptors, with K_i_ values about 1000-fold higher and inverse agonist effects at the CB_1_ and CB_2_ receptors [8]. Other receptors modulated by cannabinoid compounds include transient receptor potential family cation channels, such as transient receptor potential vanilloid 1 (TRPV1) activated by AEA [9] and CBD [10]. Among G protein-coupled receptors (GPCRs), GPR55, a rhodopsin-like class A GPCR [11], has been described to be antagonised by CBD and activated by various cannabinoids, such as CP 55,940, virodhamine and AEA, as well as by the endocannabinoid-like substance palmitoylethanolamide (PEA) [12]. In further studies, endocannabinoids such as AEA or structurally similar N-acylethanolamines, also known as endocannabinoid-like compounds such as PEA, oleoylethanolamide (OEA), stearoylethanolamide (SEA) and linoleoylethanolamide (LEA), have been described as activators of the peroxisome proliferator-activated receptor (PPAR)-α [13]. Endocannabinoid-like compounds utilise the biosynthetic and degradative enzymes of endocannabinoids but do not trigger activation of the cannabinoid receptors (for review see [14]).

The enzyme *N*-acyl phosphatidylethanolamine phospholipase D (NAPE-PLD) synthesises AEA and other *N*-acylethanolamines from membrane phospholipids. AEA is also produced via alternative biosynthetic pathways. On the other hand, 2-AG is generated by phospholipase C or by diacylglycerol lipase (DAGL) α and β (for review see [15]). AEA is catabolised by serine hydrolase fatty acid amide hydrolase (FAAH) [16], with FAAH-1 having much greater hydrolytic activity towards AEA than the FAAH-2 form, which is not expressed by rodents. The degradation of 2-AG is predominantly mediated by monoacylglycerol lipase (MAGL) or by α/β-hydrolase domain containing 6 (ABHD6) and 12 (ABHD12) [17]. Increased MAGL activity in tumour tissue is also the source of free fatty acids, which promote the growth and spread of tumours in the body through the formation of oncogenic lipids [18]. In addition, AEA and 2-AG were found to be oxidised by cyclooxygenase-2 (COX-2), leading to the corresponding prostaglandin (PG) ethanolamides and glycerol esters, respectively (for review see [14]).

In recent years, an increasing number of compounds designed to inhibit FAAH and MAGL have been tested for their preclinical potential, including antitumour activity as described later. The rationale here is to increase systemic and local endocannabinoid concentrations. Thus, shortly after the discovery of AEA degradation by FAAH [16], the same group introduced the synthetic fatty acid compound arachidonoyl trifluoromethyl ketone, which shows inhibitory properties towards FAAH with simultaneous binding affinity to the CB_1_ receptor [19]. However, this inhibitor still exhibited potent inhibitory action toward cytosolic phospholipase A_2_ (cPLA_2_) [20]. One of the first FAAH inhibitors that did not exhibit inhibition of cPLA_2_ and/or binding affinity to the CB_1_ cannabinoid receptor was *N*-arachidonoyl-serotonin (AA-5HT) [21]. This was later followed by URB597, a more potent FAAH inhibitor that even increased AEA levels in the brain, thereby exhibiting benzodiazepine-like properties [22].

A MAGL inhibitor tested in 2005 is the *N*-biphenyl carbamate URB602, which showed selectivity towards MAGL compared to FAAH inhibitor URB597 in an early attempt to establish MAGL inhibitors [23]. However, a study investigating the specificity of URB602 in measuring the inhibition of AEA hydrolysis in brain membranes found an IC_50_ value of 17 µM, while the IC_50_ value for MAGL inhibition was found to be 25 µM [24]. On the other hand, JZL184, a piperidine carbamate, proved to be a highly potent and selective MAGL inhibitor, irreversibly inhibiting the enzyme by carbamoylation of the active site catalytic serine nucleophile (Ser122). JZL184 shows IC_50_ values of 2 nM on murine and human MAGL and 25 nM on rat MAGL [25]. Later, MAGL inhibitors with lower cross-reactivity were synthesised, such as the *N*-hydroxysuccinimidyl carbamate MJN110, which showed an IC_50_ of 2.1 nM at MAGL in murine brain homogenates with concomitant lack of inhibition on AEA hydrolysis up to a concentration of 50 µM [26]. The O-aryl, O-hexafluoroisopropyl carbamate JW651 was found to be another selective inhibitor of MAGL, with an IC_50_ of 38 nM, showing less cross-reactivity with other brain serine hydrolases, such as ABHD6 (IC_50_ at 10.38 µM) and FAAH (IC_50_ > 100 µM), and inducing a 5- to 10-fold increase in brain 2-AG concentration in mice after doses of 5 to 40 mg/kg [27]. Currently, the hexafluoroisopropylcarbamate-based irreversible MAGL inhibitor ABX-1431 [28], developed in 2018, is being tested in clinical trials for its effect in, among others, neuralgias, neuropathies, motor tic disorders or Tourette’s syndrome [29]. Although appropriate clinical tests of MAGL inhibitors for efficacy and safety in relation to use as systemic cancer therapy are pending, the preclinical data presented below provide hope that such cannabinoid compounds could decisively expand the armamentarium for the treatment of tumour diseases.

It should also be mentioned that it was recently found in the distantly related roundworm *C. elegans* that JZL184 causes a prolongation of the lifespan despite the absence of a MAGL ortholog [30]. The cause of the effect of JZL184 was identified as an inhibition of the enzyme FAAH-4, which in this organism fulfils the MAGL-equivalent function of degrading endocannabinoid-related monoacylglycerides.

**Figure 1:**
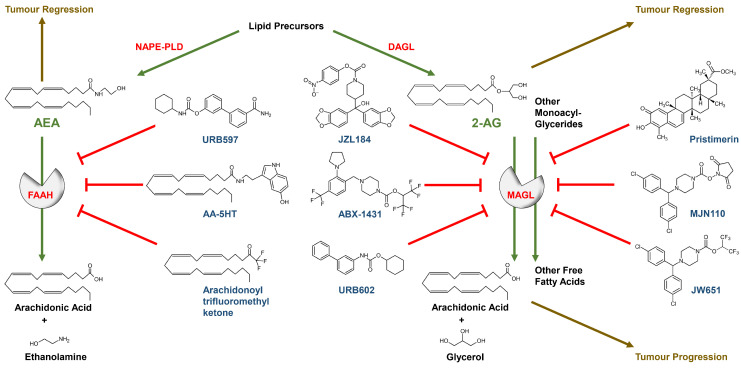
Chemical structures of selected FAAH and MAGL inhibitors, endocannabinoids and their degradation products. Green arrows: enzymatic activity; red blocking arrows: blockade of enzymatic activity; brown arrows/letters: functional influence on tumour development; red letters: enzymes; green letters: FAAH and MAGL substrates; blue letters: inhibitors of enzymes; black letters: starting compound of biosynthesis and metabolic end products of endocannabinoid degradation. For illustration purposes, only the most important enzymes are listed.

The chemical structures of the most important FAAH and MAGL inhibitors are shown together with the biochemical degradation reactions of AEA and 2-AG via FAAH and MAGL in Figure 1. 

## 2. Regulation of the Endocannabinoid System in Different Tumour Entities in Context with the Clinical Outcome of Cancer Patients

### 2.1. Regulation of Endocannabinoids in the Tumour Process

Despite many studies describing cannabinoid compounds and cannabinoid-activated receptors as mediators of anticarcinogenic effects, several investigations have reported increased activity of the endocannabinoid system in cancer tissue compared to healthy tissue and an associated unfavourable patient prognosis. However, particularly regarding the concentrations of endocannabinoids in the tumour, it is apparent that these regulations do not form a consensus of the overall profile.

Significantly elevated serum 2-AG levels have been detected, for example, in late-stage diffuse large B-cell lymphomas [31] and in biopsies from patients with endometrial carcinomas compared to healthy controls [32]. Interestingly, male patients with diffuse large B-cell lymphoma with a body mass index (BMI) ≥ 25 showed increased 2-AG levels compared to patients with a BMI < 25, while such effects were not detected in female patients [31]. Another study demonstrated increased AEA levels with almost unchanged 2-AG in colorectal carcinoma [33], whereas conversely a downregulation of AEA was observed in brain cancer tissue [34,35]. As can be seen from Table 1, endocannabinoid regulation does not correlate uniformly with tissue malignancy, the data on this are partly contradictory, and endocannabinoid levels are not yet reliable markers for tumour diseases according to current knowledge. Moreover, there are no robust studies to date linking endocannabinoid regulation to patient survival.

### 2.2. Regulation of Cannabinoid Receptors in the Tumour Process

Regarding the regulation of cannabinoid receptors in malignant tissues, similar to the regulation of endocannabinoids, studies have also shown partly contradictory results. Thereby, the majority of studies point to an upregulation of the CB_2_ receptor in tumour tissues. This is the case, for example, in non-small cell lung cancer (NSCLC) [75] (Table 1), and is consistent with other publications that have also found an association between CB_2_ receptor upregulation and cancer development and poorer survival, such as in squamous cell carcinoma of the head and neck [68], renal cell carcinoma [95] and HER2-positive breast cancer [47]. In addition, recent studies have reported that increased expression of the human epidermal growth factor 2 (HER2)-CB_2_ receptor heteromer in breast tumours is associated with lower disease-free survival in patients [48] and that lower CB_2_ levels in tumour-associated macrophages from colorectal cancer patients are associated with longer survival [57]. On the other hand, longer survival was found to be associated with higher expression of CB_2_ in hepatocellular carcinoma [69], lung cancer [76] and mobile tongue squamous cell carcinoma [83].

Analyses of CB_1_ receptor expression in cancer cells or tissues have yielded more pronounced conflicting results. An early study reported that the CB_1_ receptor in astrocytoma tissue had no dynamics associated with disease severity, while CB_2_ receptor expression in grade IV astrocytomas was higher than in lower grade tumours [37]. Another investigation concluded an association between high expression of the CB_1_ receptor in malignant tissues of patients with pancreatic cancer and poor patient prognosis, whereas CB_2_ receptor immunoreactivity did not correlate with survival [86]. Similar results have been reported for patients with prostate cancer, in whom high CB_1_ expression was associated with shorter survival [89]. In addition, high expression of CB_1_ correlated with metastasis to lymph nodes and distant organs as well as poor prognosis in esophageal squamous cell carcinoma [64]. The authors found no association with other factors such as age, sex, histologic differentiation and pathologic stage and thus concluded that immunohistochemical detection of CB_1_ could serve as a useful diagnostic marker for predicting metastases in lymph nodes and distant organs. In colorectal cancer patients undergoing surgical resection, low CB_1_ receptor expression was found more frequently in stage IV than in stage I/II or III [54]. Interestingly, stage IV patients with high CB_1_ expression in the tumours had significantly worse overall survival than patients of this stage with low CB_1_ expression, with no corresponding differences found in patients at other stages. In contrast, cannabinoid receptor regulation in lung cancer tissues was associated with increased survival in patients with high expression of CB_1_ [76]. A recent study investigating a possible function of cannabinoid receptors in the development and progression of metastases in patients with colorectal cancer found significant downregulation of CB_1_ at the mRNA and protein levels in cancer tissue compared to healthy mucosa. Interestingly, CB_1_ was also downregulated in patients with metastases in both normal mucosa and tumour tissue compared to patients without metastases, which is a possible indication that the development of metastases may be related to a reduction in CB_1_ receptor-dependent signalling pathways [58]. 

More detailed studies have shown how complex the regulation of cannabinoid receptors in tumour tissue actually is and that a simplified use of these receptor regulations as tumour markers is only possible to a limited extent. In some cancers, cannabinoid receptor regulations appear to even depend on specific molecular subsets of the cancers, such as the increased CB_1_ receptor expression in TP53-mutated Sonic Hedgehog (SHH) disease compared to other molecular categories within the medulloblastoma group [63]. In this study, none of the four molecular groups of medulloblastoma (wingless [WNT], SHH, group 3 and group 4) exhibited an association with differences in CB_2_ expression [63]. A publication focusing on gender differences in the expression of cannabinoid receptors in patients with mobile squamous cell carcinoma of the tongue reported that CB_2_ and simultaneous CB_1_/CB_2_ inductions were significantly more frequent in female patients than in male patients [83]. In the latter study, increased expression of CB_1_ in mobile squamous cell carcinomas of the tongue was associated with improved survival. A further investigation regarding the regulation of cannabinoid receptors in malignant tissue was also able to show a significant increase in CB_1_ receptors in early hepatocarcinomas [69]. Here, the Kaplan-Meyer curves revealed significant disease-free survival with high CB_1_ expression and, conversely, a significant association between low CB_1_ expression and recurrence of hepatocarcinomas. Thus, although there is a tendency for upregulation of cannabinoid receptors in cancer tissue (Table 1), this tendency cannot be consistently associated with unfavourable patient outcomes. Table 2 provides an overview of the regulation of cannabinoid receptors, FAAH and MAGL and the respective association with patient survival.

### 2.3. Regulation of Endocannabinoid-Synthesising and -Degrading Enzymes in the Tumour Process

A similar ambivalent expression pattern is currently assumed for endocannabinoid-synthesising and -degrading enzymes in malignant tissue. In one study, for example, NAPE-PLD, FAAH and MAGL were found to be downregulated in gliomas, while the expression of DAGL remained almost unchanged [34]. MAGL expression has been found to be downregulated in cancer tissue in some studies [32,34,46,49,57,74,86] but upregulated in others [18,43,53,62,71,72,73,78,82,84,92] (see Table 1).

In one study, low FAAH and MAGL immunoreactivities in cancer cells were correlated with shorter patient survival [86]. Accordingly, in this work, median survival with low FAAH levels in cancer cells was 10 months, whereas patients with moderate to strong FAAH immunoreactivity showed a median survival of 19.1 months. After resection of pancreatic cancer, patients with high MAGL levels had a median survival of 8 months, while moderate to strong MAGL staining was associated with a median survival of 21.8 months. However, this result contrasts with a report showing that MAGL worsened the prognosis of patients with hepatocellular carcinoma [71], where conversely low MAGL levels were associated with longer survival. In a further investigation on hepatocellular carcinoma, it was reported that the clinical prognosis for the group with high MAGL expression was significantly worse than for the group with low MAGL expression, in terms of overall survival times and recurrence rates [72]. In a study with 412 prostate cancer patients, higher FAAH levels were also associated with disease severity in a subgroup with intermediate but not high CB_1_ receptor levels [91]. In the latter work, higher expression of tumour epithelial FAAH was associated with a poor disease-specific survival for patients at the end stages of the disease. Another paper that focused specifically on MAGL regulation in human tumour-associated macrophages described that MAGL expression in macrophages from fresh carcinoma tissue was significantly lower than in adjacent normal tissue in colorectal cancer patients [57]. Remarkably, higher MAGL levels in tumour-associated macrophages from colorectal cancer patients were associated with better survival here, again suggesting that endocannabinoid system regulations should be interpreted with caution in terms of functional involvement in carcinogenic processes.

Furthermore, individual studies described opposite regulations of the same parameter, such as the regulation of MAGL in lung tumour tissue, which was upregulated once with patients exhibiting high MAGL expression associated with worse outcomes [78] and downregulated in malignant tissue in another study [46,74]. One reason for such discrepancies could be different patient cohorts. For example, while in Zhang et al. [78] only about 53% of the 156 patients were older than ≥60 years, the patient population studied by Liu et al. [74] was older (79% of the 34 patients ≥60 years). Finally, there are also studies that did not find an association of overall survival with FAAH expression in breast cancer patients [45] or MAGL expression in lung cancer patients’ overall survival [77]. The latter investigation, however, found the overall survival gradually reduced with increasing ABHD6 levels.

Upregulation of NAPE-PLD and FAAH mRNA has been noted in colorectal cancer tissue [33]. Elevated levels of NAPE-PLD, associated with increased AEA concentrations, have also been reported in human hepatocellular carcinoma compared to adjacent healthy tissue [70]. However, in a study with biopsies of hepatocellular carcinomas, no significant differences in NAPE-PLD mRNA were detected between tumours and non-cancerous controls [73]. The latter investigation also reported elevated 2-AG levels as a result of strong overexpression of DAGL α with less pronounced MAGL induction in hepatocellular cancer tissue; downregulation of AEA was found to be thereby dependent on higher expression levels of FAAH. In contrast to these observations, a decrease in FAAH protein expression and an increase in NAPE-PLD in cancerous versus non-cancerous endometrial tissue has recently been reported [61], resulting in increased AEA and PEA levels in cancerous endometrial tissue and in plasma from patients with endometrial carcinoma compared to volunteers with an atrophic endometrium [60].

Thus, the quantitative ratio of endocannabinoid-synthesising and -degrading enzymes to each other is crucial for the effect on endocannabinoid levels and thus tumour progression. As with endocannabinoids and cannabinoid receptors, the relationship between tumour progression and the expression of enzymes of the endocannabinoid system is complex and requires further investigation.

## 3. Systemic Effects of Cannabinoid Compounds on Different Levels of Carcinogenesis

The first studies and descriptions of tumour-regressive effects of cannabinoids date back to a 1975 publication [100], which reported that Δ^8^-tetrahydrocannabinol, THC and cannabinol suppressed tumour growth in a murine Lewis lung adenocarcinoma model, leading to a life prolongation of the experimental animals. However, relevant studies that made cannabinoid receptors a research topic as targets for systemic cancer therapy were only published from the late 1990s onwards [101].

There are now several publications showing that cannabinoid agents mediate antitumour effects via the activation of CB_1_, CB_2_ and TRPV1 or independently of these receptors (for review see [102]). In addition to directly influencing downstream signalling pathways induced via cannabinoid-triggered receptors through the application of exogenous agonists, a second strategy is to activate these receptors by increasing endocannabinoid levels at the corresponding pathological hotspots through the inhibition of endocannabinoid-degrading enzymes (for review see [14]). In this context, it is hypothesised that the endocannabinoid system, as part of an endogenous tumour defence mechanism, can counteract the growth of neoplasms and thus selective inhibitors of endocannabinoid degradation could support this tumour defence system. In addition to blocking FAAH, inhibition of MAGL as a potential antitumour strategy has also attracted the attention of scientists in recent years, as it not only induces anticarcinogenic effects by activating cannabinoid receptors via 2-AG, but also reduces a number of tumour-promoting fatty acids (Figure 1) that would contribute to tumour growth via various mechanisms when MAGL is overactive (for review see [14,102]).

In the following, the effects of cannabinoids and subsequently of inhibitors of endocannabinoid degradation on the key events of tumour development and spread are described respectively. In the case of the cannabinoids presented for reasons of comparison, only selected studies are mentioned.

### 3.1. Cancer Cell Proliferation and Viability

#### 3.1.1. Effect of Cannabinoids on Cancer Cell Proliferation and Viability

Publications on growth-inhibiting effects of cannabinoids have accumulated over the last two decades. Studies from the beginning of the millennium focused on the mechanisms leading to cannabinoid-induced apoptosis and cell cycle arrest of cancer cells. Later, some extensive studies showed a connection between cannabinoid-induced autophagy and subsequent apoptosis.

A seminal paper on this topic was published in 2000, showing that THC and the synthetic cannabinoid WIN 55,212-2 induced growth inhibition of glioma xenografts in Wistar rats and in Rag2-/- mice [103]. The crucial mechanism identified was a sustained accumulation of ceramide as a second messenger leading to cell death of glioma cells, which was later confirmed for *R*(+)-methanandamide (Met-AEA)-induced apoptosis of neuroglioma cells [104,105]. Meanwhile growth inhibitory effects of various cannabinoid compounds have been reported in numerous studies. In this context, protein kinase B (Akt) inhibition has been repeatedly described as a key event in the growth inhibitory effects of cannabinoids on various types of tumour cells, as shown for instance for CB_1_ receptor-dependent WIN 55,212-2-induced cell cycle arrest in melanoma cells [106], CB_1_ receptor dependent HU210- and THC-induced apoptosis of rhabdomyosarcoma cells [97] or CB_2_ receptor-dependent THC- and JWH-133-induced apoptosis of breast cancer cells [107]. In this context, a number of cell cycle regulators have been found to be affected by cannabinoids [42,108].

In addition, recent studies have shown that the induction of autophagy is associated with these apoptotic effects and is involved in the toxicity of cannabinoid compounds to cancer cells. This was first demonstrated by Salazar et al. [109], who reported that THC induces autophagy in glioma cells via CB_1_ receptor- and ceramide-dependent phosphorylation of eukaryotic translation initiation factor 2α (eIF2α) and subsequent endoplasmic reticulum stress, induction of the stress-associated transcriptional coactivator p8 and upregulation of tribbles pseudokinase 3 (TRB3). These regulations eventually lead to autophagy via inhibition of Akt and mTORC1 (mammalian target of rapamycin complex 1). In this publication, autophagy represents a mechanism that ultimately leads to apoptosis and cell death of tumour cells. In another study with human hepatocellular carcinoma cells, it was shown that autophagy induction occurs through two different mechanisms, both of which also lead to tumour cell death. Here, THC and the CB_2_ agonist JWH-015 also induced TRB3 upregulation and subsequent inhibition of the Akt/mTORC1 axis, but a second pathway involved cannabinoid-induced activation of Ca^2+^/calmodulin-activated kinase kinase β (CaCMKKβ) with a causally linked downstream stimulation of adenosine monophosphate-activated kinase (AMPK), which ultimately caused autophagy [110]. The same authors later further demonstrated upregulation of PPARγ as a link between autophagy and apoptosis in hepatocellular carcinoma cells treated with THC or the selective CB_2_ agonist JWH-015 [111]. Further evidence for autophagy inductions was found for THC in melanoma cells [112] and for CBD in various cancer entities, such as glioma tumour cells [113], glioma stem-like cells [114], breast cancer [115], lymphoblastic leukaemia [116] and head and neck squamous cell carcinoma cells [117].

#### 3.1.2. Effect of FAAH and MAGL Inhibition on Cancer Cell Proliferation and Viability

Consistent with the concept that an increase in endogenous endocannabinoid levels also counteracts cancer processes in the body, inhibitors of endocannabinoid-metabolising enzymes revealed an antiproliferative effect on cancer cells. One of the early pioneering studies in this field showed that arachidonoyl trifluoromethyl ketone, an inhibitor of the major AEA-degrading enzyme FAAH, enhanced the antiproliferative effect of exogenously added AEA on breast cancer cell lines [101]. Subsequently, several publications confirmed the anticancer effect of inhibitors of FAAH. For example, AA-5HT, another FAAH inhibitor, was described to cause growth inhibition of thyroid cancer xenografts [118] and to reduce tumour development in an in vivo model of colon carcinogenesis [119]. The inhibition of tumour cell growth by FAAH inhibition was later also shown in melanoma cells. Thus, a study demonstrated concentration-dependent apoptosis of melanoma cells after incubation with AEA, which was enhanced by inhibition of FAAH and attenuated by blockade of COX-2 or lipoxygenase (LOX), suggesting involvement of eicosanoid metabolism in this endocannabinoid effect [120]. In another study, antiproliferative effects on melanoma cells were described for PEA and 2-AG in addition to AEA [121]. This work focused primarily on the antiproliferative effect of PEA and its enhancement due to FAAH inhibition, which was finally also confirmed in vivo using a murine xenograft model [121]. In another study, FAAH inhibition in combination with 2-methyl-2′-fluoro-anandamide (Met-F-AEA) was shown to lead to cell cycle arrest in the G0/G1 phase and apoptosis in lung cancer cells, mediated by a reduction in epidermal growth factor receptor (EGFR) activation and downstream signalling pathways [122]. In addition to the aforementioned tumour regressive findings on AA-5HT, which caused a reduction in tumour growth alone without the addition of exogenous FAAH substrates [118,119], there are also other in vivo studies with FAAH inhibitors in which such effects were not observed. For example, no reduction in tumour development was demonstrated when URB597 alone was administered every third day for three weeks at a dose of 1 mg/kg in a lung tumour model [122] or daily for six days at a dose of 10 mg/kg in a melanoma model [121]. Similarly, using a lung tumour xenograft model, it was shown that both URB597 and AA-5HT did not significantly affect tumour growth when the compounds were administered alone at a dose of 10 mg/kg every 72 h for 28 days [123].

In recent years in particular, data have accumulated supporting the inhibition of MAGL as a promising concept for inhibiting cancer progression. On the one hand, inhibition of the key 2-AG-degrading enzyme MAGL by JZL184 exhibited positive effects on the quality of life of experimental animals in preclinical studies, such as reduced cachexia in a mouse model for bone cancer [92] and in lithium chloride-induced vomiting in tree shrews [124]. In addition, inhibition of MAGL has also been found to induce systemic anti-cancer effects, either by affecting the endocannabinoid system or by modulating endogenous lipids. In a first of these studies with breast, ovarian and melanoma cancer cells, a decrease in free fatty acids due to MAGL inhibition was considered to be the main cause of the anti-cancer potential of MAGL inhibition [18]. Consistent with this hypothesis, aggressive melanoma cells in which MAGL was knocked down by small hairpin (sh) RNA showed decreased tumour growth, which was completely counter regulated in mice fed a high-fat diet, associated with increased free fatty acid content in the tumours. Knockdown of MAGL or treatment with the MAGL inhibitor JZL184 also caused inhibition of prostate cancer xenograft growth [125]. Here, the authors found partial reversal of tumour growth defects observed with MAGL shRNA-transfected PC3 cells by treatment of mice with a high-fat diet or a CB_1_ receptor antagonist, and fully reversal by pre-treatment with both regimes. The importance of lipid modulation by MAGL inhibition was further underscored by a recent study showing that JZL184-mediated blockade of oleic acid-stimulated proliferation of glioblastoma cells occurs via modulation of triglyceride metabolism and not via changes in cannabinoid receptor signalling pathways [126]. A tumour-regressive effect has also been reported for JZL184 in a murine xenograft model with MAGL-overexpressing hepatocellular cancer cells [71]. The growth inhibitory effect of MAGL inhibition by knockdown or treatment with JZL184 was further corroborated in a colon cancer cell xenograft model and associated with downregulation of cyclin D1 and B-cell lymphoma 2 (Bcl-2) [53]. Other studies have confirmed these findings and additionally found an increase in Bcl-2-associated X protein (Bax) in response to treatment with JZL184 in colorectal cancer cells [127]. Knockdown of MAGL by transfection with a shRNA likewise inhibited lung cancer cell proliferation via inhibition of cyclin B1 and cyclin D1 expression [78]. A reduction in the expression of cyclin D1 and Bcl-2, which was associated with a reduction in cell cycle progression after treatment with JZL184 and by small interfering (si) RNA-mediated MAGL inhibition, was finally also demonstrated in endometrial cancer cells [62]. However, not all studies could support these positive effects of MAGL knockdown on cancer progression. Accordingly, a recent publication found that inhibition of MAGL enhances rather than impairs cancer progression in mice [74]. The authors obtained their results in MAGL knockout mice, which showed a higher incidence of neoplasia in multiple organs with splenomegaly and particularly promoting effects on lung cancer progression. MAGL deficiency was associated with increased EGFR expression and phosphorylation, as well as increased activation of p42/44 mitogen-activated protein kinase and Akt, and induction of COX-2 and tumour necrosis factor α. 

The in vitro evidence on the effects of MAGL inhibition on tumour cell viability is ambiguous. In a recent study, JZL184 at concentrations between 0.01 and 10 µM was shown to neither significantly reduce viability and proliferation of lung cancer cells under both serum-containing and serum-free conditions [128]. The same work also demonstrated that JZL184 did not significantly alter the colony forming properties of lung tumour cells in this concentration range. On the other hand, MAGL siRNA led to a slight but significant decrease in viability [128]. These data are in line with observations in melanoma, ovarian and breast cancer cells, where knockdown of MAGL via transfection of shRNA but not pharmacological inhibition of MAGL with JZL184 decreased cellular viability, implying that long-term inhibition of MAGL may be necessary for this effect [18]. Another study found that a 3-day treatment of prostate cancer cells with JZL184 resulted in an increase in cell density under basal conditions but a decrease in epidermal growth factor (EGF)-induced proliferation [129]. No effect of JZL184 was observed on the viability of neuroblastoma cells after an incubation of 3 days [130]. Here, the authors observed a moderate toxic effect on neuroblastoma cells only induced by the MAGL inhibitors methyl arachidonyl fluorophosphonate (MAFP) and CAY1049 or in the presence of 2-AG. On the other hand, cytotoxic effects of JZL184 have been observed in endometrial carcinoma [62], colorectal cancer [53] and hepatocellular carcinoma cells [71]. Accordingly, the effect of MAGL inhibitors seems to depend on the particular conditions and cell line as well as on the duration of action and the concentration used. 

Among the compounds known to affect MAGL activity, the plant triterpenoid quinone methide pristimerin has attracted attention in recent years as a reversible MAGL inhibitor [131]. Inhibition of cancer growth by pristimerin treatments was demonstrated in a murine lung cancer xenograft model [132]. In addition, pristimerin was shown to induce apoptosis, autophagy and cell cycle arrest in human breast cancer cells [133]. However, an effect on the endocannabinoid system as the basis of the anticancer properties of pristimerin was not investigated in the aforementioned works.

In general, there are no studies on the influence of MAGL inhibition on the induction of autophagy in tumour cells. However, it should be noted that the above studies overwhelmingly support the hypothesis that endocannabinoid-degrading enzymes, especially MAGL, are key parameters in the endogenous control of cancer growth. This makes these enzymes promising targets for innovative pharmacotherapeutic options that could benefit cancer patients.

### 3.2. Cancer Cell Invasion and Metastasis

#### 3.2.1. Effect of Cannabinoids on Cancer Cell Invasion and Metastasis

Many investigations have addressed anti-invasive and antimetastatic effects of various cannabinoid compounds, as summarised elsewhere [102]. Early studies on this topic showed that 2-AG exerts a CB_1_-dependent anti-invasive effect on prostate cancer cells [134]. Later, anti-invasive effects were confirmed for AEA in glioma [135] and lung cancer cells [123] as well as for the endocannabinoid derivatives Met-F-AEA in human breast cancer cells [136] and for Met-AEA in cervical and lung cancer cells [137]. Both endocannabinoid derivatives caused anti-invasive effects via CB_1_ receptor activation. In the case of Met-AEA, the CB_2_ receptor and TRPV1 additionally contributed to the inhibition of invasion in the latter study, with THC exerting an invasion-inhibiting effect via CB_1_ and CB_2_ in the same system [137]. Thereby, both cannabinoids mediated the inhibition of invasion via an induction of the tissue inhibitor of metalloproteinase-1 (TIMP-1), which could later also be confirmed for CBD in cervical and lung cancer cells [138]. Another study found a causal relationship in lung cancer cells between THC-, CBD- and Met-AEA-induced invasion inhibition and the induction of the expression of intercellular adhesion molecule-1 (ICAM-1), which acts as an upstream regulator of TIMP-1 [139]. In the same work, ICAM-1 was also shown to mediate the antimetastatic effect of CBD in athymic nude mice. Further proteolytic regulations involved in anti-invasive cannabinoid action include downregulation of matrix metalloproteinase (MMP)-2 by THC in glioma cells [140], downregulation of MMP-2 and -9 in hepatocellular carcinoma cells treated with the CB_2_ receptor agonist CB65 and the CB_1_ receptor agonist arachidonyl-2′-chloroethylamide (ACEA) [141] and downregulation of plasminogen activator inhibitor-1 (PAI-1) as underlying mechanism of CBD-induced inhibition of lung cancer cell invasion [142]. Moreover, reduced MMP-2 release from cancer-associated fibroblasts after treatment with WIN 55-212.2 led to reduced invasion of prostate cancer cells, suggesting that cannabinoids inhibit invasion not only by direct action on tumour cells [143].

Besides the regulation of proteolytic enzymes as the underlying mechanism of the anti-invasive effect of cannabinoids, other studies showed that the anti-invasive mechanism of CBD also relies on downregulation of the inhibitor of basic helix-loop-helix transcription factors, Id-1, in breast cancer [144] and brain tumour cells [145]. The antimetastatic effect on breast cancer cells demonstrated for CBD in a mouse model [146] was shown in a later work to be reversible by ectopic expression of Id-1 in breast cancer cells [147], so that the antimetastatic action of CBD can also be directly linked to Id-1 downregulation.

#### 3.2.2. Effect of FAAH and MAGL Inhibition on Cancer Cell Invasion and Metastasis

Some recent publications have revealed that regulation of the endocannabinoid system through inhibition of endocannabinoid-degrading enzymes has a profound effect on tumour cell invasion and metastasis. This involves some of the mechanisms that have also been demonstrated for the synthetic and phytocannabinoids. For example, CB_2_- and TRPV1-mediated induction of TIMP-1 was confirmed as the mechanism of anti-invasive action for the FAAH inhibitors AA-5HT and URB597, as well as for FAAH siRNA in human lung cancer cells [123]. In the latter study, beyond the in vitro results, administration of AA-5HT and URB597 and the FAAH substrates AEA, 2-AG, OEA and PEA to athymic nude mice was reported to have an inhibitory effect on metastatic infiltration of the lung by previously intravenously injected lung carcinoma cells.

In addition, there are other anti-invasive mechanisms that have been shown to be specific to the inhibition of endocannabinoid degradation. In this context, the role of hydrolysis of monoacylglycerols and thus the function of MAGL in cancer metastasis has been intensively researched. In a pioneering comprehensive investigation [18], shRNA-mediated silencing of MAGL was shown to inhibit migration and invasion of the melanoma cell line C8161 and the ovarian cancer cell line SKOV3, with the migration reduced by shRNA being reversed in both cell lines by treatment with fatty acids. In a follow-up study with prostate cancer cells, inhibition of cancer cell migration after MAGL inhibition by JZL184 or MAGL shRNA was partially reversed by the addition of fatty acids or CB_1_ receptor antagonists and completely reversed by the combination of both substances [125]. Therefore, activation of the CB_1_ receptor by the increase in 2-AG and a decrease in tumour-promoting free fatty acids, both induced by MAGL inhibition, appear to produce these effects on prostate cancer cells. Other work with hepatocellular carcinoma cells showed increased invasion when the cells overexpressed MAGL by knock-in and, conversely, decreased invasion when MAGL was knocked out or inhibited by JZL184 [71]. Therefore, the authors considered an increase in PGE_2_ and lysophosphatidic acid as promoters for increased proliferation and invasion as the mechanism for this effect. In another study, using an shRNA approach, it was found that inhibition of MAGL expression was associated with reductions in lung cancer cell invasiveness, lung cancer xenograft growth and lung cancer metastasis [78]. In this context, the authors described a downregulation of MMP-14 that mediated a reduction in invasiveness triggered by MAGL knockdown. Another recent study addressing the role of MMP-dependent proteolytic action in the context of the anti-invasive properties of MAGL inhibition revealed CB_1_ receptor-dependent anti-invasive and antimetastatic effects of JZL184 on human lung cancer cells [128], with anti-invasion mediated by increased TIMP-1 expression. In the latter study, a TIMP-1-dependent anti-invasive effect was confirmed for the MAGL substrate 2-AG. In addition, invasion inhibitory effects associated with increased TIMP-1 expression were observed for the MAGL inhibitors JW651 and MJN110 or when cells were transfected with MAGL siRNA. Finally, anti-invasive effects of JZL184 have also been shown for colorectal cancer [53] and hepatocellular carcinoma cells [71].

In further work, different immunodeficient mouse models were used to investigate the effects of JZL184 on bone cancer metastasis by intracardiac injection of osteotropic human prostate and breast cancer cells and by paratibial injection of human osteosarcoma cells [92]. The latter system was complemented by a murine osteosarcoma cell line as syngeneic model. Here, JZL184 inhibited skeletal tumour growth in all applied systems reduced bone metastasis of breast and prostate cancer cells, and inhibited the spread of osteosarcoma cells to the lung. These results are consistent with another work reporting that knockdown of MAGL reduced invasion of highly metastatic nasopharyngeal carcinoma cells and decreased the proportion of popliteal lymph node metastases in vivo [84]. The latter study also described that MAGL expression and metastatic potential of nasopharyngeal carcinoma cells were associated with a decrease in epithelial-mesenchymal transition (EMT) markers. Thus, MAGL-overexpressing cells showed increased vimentin and snail levels and decreased E-cadherin levels, which was reversed by knockdown of MAGL [84]. Inhibition of migration by JZL184 was also associated with inhibition of EMT, i.e., downregulation of vimentin and Snail and upregulation of E-cadherin in another study with colon cancer cells [127]. Consistent with the notion of MAGL as a proinvasive parameter, further investigation revealed that MAGL-overexpressing hepatocellular carcinoma cells exhibited higher invasiveness, which was causally linked to increased expression of nuclear factor-κB p65, which regulates downstream markers of EMT [72].

### 3.3. Tumour Angiogenesis

#### 3.3.1. Effects of Cannabinoid Compounds on Tumour Angiogenesis

In several publications, a reduction of neovascularisation of xenograft tumours in mice treated with cannabinoids has been demonstrated, sometimes only as an additional finding, as described elsewhere (for review see [102]). In terms of possible mechanisms, early studies indicated that cannabinoids downregulate VEGF (vascular endothelial growth factor) and angiopoietin-2 (Ang-2) in mouse skin [148] and glioma tumours [149], with additional inhibition of MMP-2 formation described in glioma tumours. Later, the same group found modulation of several hypoxia-related angiogenesis markers by the CB_2_ receptor agonist JWH-133 in mouse gliomas, including hypoxia-inducible factor-1α, connective tissue growth factor, midkine, Id-3, heme oxygenase-1 and, in addition to Ang-2, its receptor tyrosine kinase, with immunoglobulin-like and EGF-like domains 1 (Tie-1) [150].

Other studies focused on the interaction of cancer cells and their associated microenvironment. For example, an investigation with conditioned media of CBD-, THC-, Met-AEA- and JWH-133-treated lung cancer cells showed an inhibition of tube and sprout formation as well as endothelial cell migration [151]. Here, the authors showed that the increased release of TIMP-1 by cannabinoids from lung tumour cells mediates inhibition of the angiogenic properties of endothelial cells in addition to the anti-invasive effect described above, suggesting a dual anticancer mechanism of cannabinoid-induced TIMP-1 expression. Another report addressing the interplay between the immune system and tumour cells showed that ACEA and JWH-133 inhibited lipopolysaccharide (LPS)-induced VEGF-A release from human neutrophils, which was associated with the decrease in the corresponding LPS-induced angiogenic capacities of bovine aortic endothelial cells [152].

#### 3.3.2. Effects of FAAH and MAGL Inhibition on Tumour Angiogenesis

Regarding the effects of endocannabinoids, it was found that the conditioned medium of AEA-treated breast cancer cells inhibited the proliferation of endothelial cells, which was attributed to the downregulation of factors involved in angiogenesis, such as leptin, thrombopoietin and VEGF [153]. Interestingly, there are no data on the effects of FAAH inhibition on the interaction between cancer cells and endothelial cells, as has been reported for other cannabinoids. In the case of MAGL, one study found that the MAGL inhibitor URB602 reduced the growth of colon carcinoma xenografts, which was associated with reduced VEGF and fibroblast growth factor (FGF)-2 expression [154]. In the same work, URB602 reduced colon carcinogenesis in a murine model of azoxymethane-induced preneoplastic lesions, polyps and tumours in the colon, with the authors suggesting this effect to be dependent on the anti-angiogenic properties of URB602. Thus, MAGL inhibition suppressed FGF-2-induced proliferation and migration of endothelial cells. Overall, these results suggest a previously neglected effect of MAGL inhibition on cancer development and progression that should receive increased attention in future studies.

Finally, it was reported that the natural MAGL inhibitor pristimerin reduced sonic hedgehog (Shh)-induced angiogenesis by inhibiting VEGF receptor phosphorylation in endothelial cells [132]. However, a contribution of MAGL inhibition to this anti-angiogenic effect was not investigated here. 

### 3.4. Tumour-Immune Interactions

#### 3.4.1. Effect of Cannabinoid Compounds on Tumour-Immune Interactions

An impact of cannabinoids on the immune system and thus on its components that influence tumour growth has been discussed for a long time. Indeed, cannabinoids greatly affect immune cells, as recently reported using single-cell transcriptome mapping, where THC altered a variety of genes involved in immune response, cytokine production, cell proliferation and apoptosis of immune cells [155]. A further recent study performed on pancreatic cancer cells indicated an inhibitory effect of THC and CBD on the expression of programmed death ligand 1 (PD-L1), a key target of immune checkpoint blockade, through an upstream reduction in the expression and phosphorylation of activated p21 kinase 1 (PAK1) [156]. Finally, another recent paper demonstrated an inhibitory effect of the CB_1_ agonist ACEA and the CB_2_ agonist JWH-133 on VEGF release from neutrophils [152]. Further exemplary studies have shown a stronger tumour-regressive effect of the synthetic cannabinoid WIN 55,212-2 in immunocompetent compared to immunodeficient mice [106], a tumour-regressive effect of THC on melanomas associated with reduced infiltration of macrophages and neutrophils [157], and increased cancer cell lysis by lymphokine-activated killer cells after treatment of lung tumour cells with CBD, THC and Met-AEA [158].

However, there are also results showing that THC accelerates tumour implant growth by inhibiting antitumour immunity via a CB_2_ receptor-mediated, cytokine-dependent pathway in Lewis lung carcinoma and alveolar carcinoma models of immunocompetent mice [159]. In another study using breast cancer cells expressing low levels of cannabinoid receptors, THC was found to increase tumour growth and metastasis due to inhibition of the specific antitumour immune response in vivo. Accordingly, THC induced the profile of Th2 polarisation, resulting in an increase in Th2-associated cytokines and a downregulation of Th1-related cytokines [160]. Finally, another investigation showed that the CB_2_ receptor-activating effect of cannabinoids attenuates its own antitumour effect by promoting M2 polarisation of microglia/tumour-associated macrophages (TAMs), causing a proangiogenic effect of M2 microglia [161].

#### 3.4.2. Effect of FAAH and MAGL Inhibition on Tumour-Immune Interactions

Accumulating data suggest that the endocannabinoid system acts as an important regulator of tumour immune defence through multiple mechanisms. For example, the involvement of the endocannabinoid system in the immune response of tumours was recently highlighted by the finding that the FAAH inhibitor URB597 may enhance the immune surveillance of human hepatocellular carcinoma cells [162]. Specifically, URB597 via induction of TIMP-3 expression was found to inhibit proteolytic shedding of the proteins major histocompatibility complex class I polypeptide-related sequence A (MICA) and B (MICB) on the surface of cancer cells, thereby ensuring binding of these proteins to the natural killer group 2D (NKG2D) receptor on the surface of cytolytic lymphocytes and subsequent killing of tumour cells. 

Likewise, MAGL has become the focus of tumour-immune interactions in recent work. Using inoculated and genetic cancer models, one study demonstrated that MAGL is oppositely regulated in cancer tissues and tumour-associated macrophages (TAMs) [57]. In this context, the authors reported an interaction between the CB_2_ receptor and Toll-like receptor 4 (TLR4) in the membrane of TAMs. Thus, upregulation of MAGL in macrophages and associated decreased levels of 2-AG led to unloading of TLR4 signalling via an interaction with the CB_2_ receptor. At the same time, overexpression of MAGL resulted in increased levels of free fatty acids, leading to activation of TLR4 signalling and M1 polarisation of TAMs. Lipid accumulation in TAMs thereby promotes CB_2_/TLR4-dependent macrophage activation, inhibition of CD8+ T cells and thus tumour progression. On the other hand, decreased MAGL activity in macrophages in the tumour microenvironment resulted in activation of CB_2_ receptor binding and inactivation of TLR4 via increased 2-AG concentration. The inhibition of MAGL activity in TAMs accordingly led to growth and metastasis of cancer cells in the tumour microenvironment. Consistently, in a mouse model with MAGL-overexpressing myeloid cells, cancer growth in experimental colorectal carcinomas was slower than in the wild type [57].

**Figure 2:**
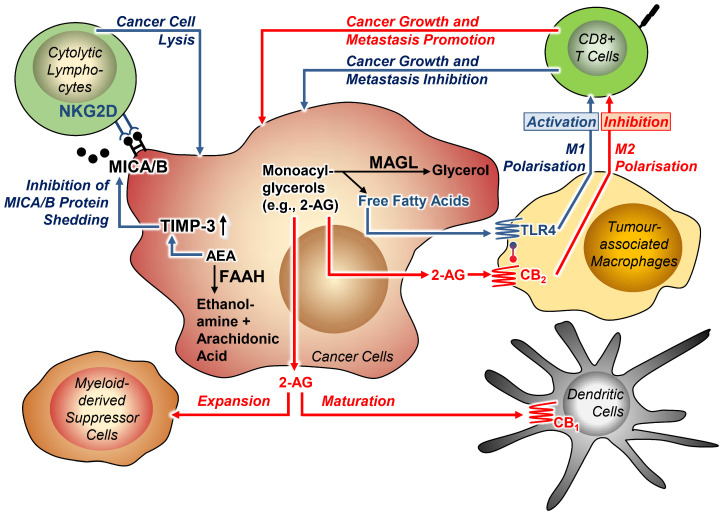
Effects of MAGL and FAAH on tumour-immune interactions. Blue arrows indicate benign effects, red arrows indicate effects that cause cancer progression. Lines with circles at the ends indicate receptor interaction. All abbreviations are explained in the text.

Another comprehensive study on this topic also addressed the role of MAGL in the interplay between cancer and immune cells and found that arsenite resistance protein 2 (ARS2) transcriptionally upregulates MAGL and thereby regulates the self-renewal and tumourigenicity of glioma stem cells via increased synthesis of PGE_2_ [163]. Consistent with the in vitro findings here, MAGL-specific shRNA-expressing lentivirally infected glioblastoma stem cells orthotopically injected into nude mice showed less pronounced xenograft growth and the corresponding mice survived significantly longer than the mice receiving cells infected with the shRNA control construct. Thus, JZL184 increased the survival of the xenografted mice in association with a downregulated M2-like signature of TAMs. Figure 2 summarises the highlights of the descriptions. Finally, it is important to note that in one study, administration of JZL184 had no effect on tumour burden in bone and spleen of immunocompetent female C57BL/6KalWRij mice that had received an injection of the murine multiple myeloma cell line 5TGM1 via the tail vein [164]. 

## 4. Conclusions and Perspectives

Many of the preclinical results listed here give hope for cannabinoids as a possible additional approach for pharmacotherapeutic interventions in cancer. According to studies in past years, FAAH and MAGL inhibitors also show preclinical tumour-regressive effects. Recent investigations also highlight that MAGL inhibitors could be promising anticancer drugs in terms of their antimetastatic properties [78,92,128]. In addition, a dual antitumour mechanism of MAGL inhibition may prove beneficial, consisting of inhibiting the degradation of antitumour 2-AG while also preventing the MAGL-dependent formation of free fatty acids that promote tumour growth and spread. Regarding the translational aspect, where cannabinoid compounds would certainly be combined with other chemotherapeutic agents, there are a number of preclinical reports indicating that cannabinoids, especially CBD and THC, enhance the chemotherapeutic effects of established cytostatic treatments in cancer (for review see [165]). However, there are currently no data on potential synergies between FAAH or MAGL inhibitors and classical chemotherapies or immunotherapies, particularly related to systemic anticancer effects. Considering that cannabinoids are already used clinically to treat various chemotherapy-induced side effects, such as nausea and vomiting (for review see [166]) as well as pain [167], this could additionally lead to benefits for cancer patients. An analgesic effect has also been reported for FAAH and MAGL inhibitors as well as for dual inhibitors of both enzymes [168]. Furthermore, a recent study has shown that the MAGL inhibitor MJN110 can completely reverse paclitaxel-induced allodynia in a mouse model of chemotherapy-induced neuropathy [169]. Accordingly, combination studies with classical chemotherapeutic agents should also be conducted for these blockers of endocannabinoid degradation, looking at a possible synergistic effect on tumour growth and spread as well as a possible reduction of chemotherapy-typical side effects. 

From the studies described above, on the regulation of the endocannabinoid system in cancer, a tendency toward upregulation of CB_2_ and CB_1_ receptors in tumour tissue can be inferred. However, these parameters do not represent reliable superior tumour markers. This is mainly due to such observations it always being necessary to distinguish exactly which cells (tumour tissue or immune cells), which degree of development of carcinogenesis and which molecular tumour subtype are present in order to derive a prognostic property from the respective parameters. Similarly, no clear indications of suitability as tumour markers can currently be derived for FAAH and MAGL.

While cannabinoids (THC, nabilone (synthetic THC analogue), nabiximols (standardised extract of *Cannabis sativa* L. with an approximate 1:1 ratio of THC and CBD), CBD) are already used therapeutically, several FAAH inhibitors and one MAGL inhibitor are still in phase II testing (for review see [170]). In the case of the MAGL inhibitor ABX-1431 investigated for use in Tourette’s syndrome, no major adverse events were described. However, preclinical studies indicate that MAGL inhibitors require a carefully designed dosing schedule to prevent CB_1_ receptor-dependent side effects. Here, animals treated with the dual FAAH and MAGL inhibitor, JZL195 or the MAGL inhibitor JZL184, showed hyperreflexia in the bar test, known as “popcorning”, and hypomotility in an open field test [168]. However, JZL184, like the FAAH inhibitor PF-3845, did not cause marked catalepsy in animals. The hypomotility typical of THC was also ruled out for FAAH inhibitors in another study [171]. On the other hand, THC-like psychoactive effects, such as catalepsy and THC-like drug discrimination reactions, were induced with dual inhibition of FAAH and MAGL [168]. Furthermore, the disaster of the clinical trial with the FAAH inhibitor BIA 10-2474 [172] clearly showed the importance of consistent preclinical selectivity testing to exclude off-target activities. Other cannabinoid-typical side effects on the cardiovascular system [173] as well as the increased risk of liver fibrosis associated with CB_1_ receptor activation [174] should also be critically evaluated.

However, the greatest challenge lies in the translational leap from the thus far successful preclinical oncological testing of cannabinoids or inhibitors of endocannabinoid degradation into the clinic. Current studies are limited to a few investigations, such as a pilot study from 2006 that showed that intracranially administered THC was safe in glioblastoma patients [175]. Another exploratory, randomised, placebo-controlled phase 1b clinical trial in patients with recurrent glioblastoma multiforme included 21 patients, 12 of whom were randomised to THC and CBD in addition to dose-intensive temozolomide, with a 1-year survival 83% for nabiximols- and 44% for placebo-treated patients [176]. 

There are a number of clinical trials registered with the United States National Library of Medicine that address the effects of cannabinoids in cancer patients beyond palliative effects, such as pain relief. These studies include an open-label, single-group phase I/Ib intervention study with 18 participants on the safety of CBD in patients with biochemically recurrent prostate cancer [177], monitoring adverse events leading to dose-limiting toxicity of CBD treatment, as well as prostate specific antigen (PSA) and testosterone level determination as an indication of biochemical response. Another clinical trial is a randomised, open-label, interventional phase III study with 160 participants on the effect of combination therapy in patients with early breast cancer using mifepristone, tamoxifen, retinoic acid and CBD as a selective CYP2D6 inhibitor to enhance the effect of tamoxifen [178]. Here, the expression of cytokeratin 5 is measured to identify a therapy-resistant subpopulation of breast cancer cells that show quantitatively increased cancer stem cell characteristics. Other parameters to be investigated include pathological complete response and concentrations of estradiol, progesterone, retinoic acid and tamoxifen in breast tissue and plasma, as well as tumour markers in serum compared to baseline. Another registered, yet to be recruiting clinical trial aims to determine the maximum tolerated dose and the type and number of adverse events occurring during treatment with a combination of THC and CBD [179]. The secondary outcome to be determined in this study is the antitumour activity of THC/CBD treatment in combination with temozolomide and radiotherapy, as well as overall survival and progression-free survival.

In summary, clinical studies need to clarify which combinations of chemotherapeutic agents with cannabinoids or, in perspective, inhibitors of endocannabinoid degradation are useful for cancer patients. To provide this development with the opportunity to succeed, uncertified products derived from marijuana should not continue to be marketed under the pretext of curing cancer [180]; this would be the second step before the first, which could jeopardise the potential use of cannabinoids in tumour diseases.

## Figures and Tables

**Table 1:** Regulation of endocannabinoids, cannabinoid receptors, FAAH, MAGL and NAPE-PLD in tumour tissues (in a few labelled cases serum/plasma) from cancer patients.

Tumour Type	AEA	2-AG	CB_1_	CB_2_	FAAH	MAGL	NAPE-PLD	Commentary	Ref.
Acute myeloid leukemia				↑					[36]
Astrocytomas			↔	↔				refers to differences between human astrocytomas of low (grades I–II) and high (grade III) malignancy and differences between pure astrocytomas and mixed oligoastrocytomas	[37]
			↔	↔					[38]
			↔	↑					[39]
	↓	↑	↔	↑	↓	↓	↓		[34]
			↑					higher expression in pediatric low-grade gliomas of spontaneous involution/stable status than in relapse	[40]
			↔	↑				refers to benign juvenile pilocytic astrocytomas	[41]
B-cell lymphoma		↑						refers to analyses of serum from male patients with a body mass index (BMI) ≥ 25 compared to male patients with a BMI < 25	[31]
Breast cancer				↑					[42]
						↑		ductal breast cancer	[43]
				↑				immunohistochemical staining revealed 58% CB_2_ receptor positive samples in 82 patients without comparison to healthy tissue	[44]
					↑			regulation not associated with disease-specific survival and recurrence-free survival, but with high number of lymph node involvement	[45]
						↓			[46]
				↑				HER2-positive breast cancer	[47]
				↑				HER2-CB_2_ heteromers	[48]
						↓			[49]
Colorectal cancer	↑	↑							[50]
			↓	↑					[51]
			↓	↔					[52]
Colorectal cancer						↑			[53]
			↓					proportion of low CB_1_ expression significantly higher in stage IV than in stage I/II or III cancer	[54]
						↓		degradation or loss of MAGL expression detected in 60 of 101 cases with colon cancer and in 9 of 18 cases with rectal cancer (from pooled data of different methods)	[46]
	↑	↔	↑	↔	↑		↑		[33]
				↑					[55]
			↓					CB_1_ mRNA levels significantly reduced in TNM stage I tumours; CB_1_ mRNA levels, however, increase with greater disease severity	[56]
				↑		↓		refers to tumour-associated macrophages	[57]
			↓					additional finding: significant downregulation of CB_1_ in patients with metastases, both in normal mucosa and tumour tissue compared to patients without metastases	[58]
Endometrial cancer		↑		↑		↓			[32]
						↓			[46]
			↑	↑					[59]
	↑								[60]
					↓		↑		[61]
						↑			[62]
Ependymoma			↑	↔				C11orf95 subtype (C11orf95 fusion-positive ependymoma, formerly named EPN_RELA, high-risk brain cancer in children) with higher CB_1_ receptor expression than the PFA subtype of ependymoma (posterior fossa type A ependymomas); CB_2_ receptor expression was similar in both subtypes	[63]
Esophageal squamous cell carcinoma			↑						[64]
Gastric cancer						↓			[46]
			↑						[65]
Glioblastoma	↓							refers to analysis of only one glioblastoma sample	[35]
			↔	↑				refers to differences between glioblastoma (grade IV) and human astrocytomas (grades I–II and III)	[37]
	↑	↑							[66]
			↔	↔					[38]
			↔	↑					[67]
			↓	↑					[39]
	↓	↑	↑	↑	↓	↓	↓		[34]
			↔	↑					[41]
Head and neck squamous cell carcinoma				↑					[68]
Hepatocellular carcinoma			↑	↑				early hepatocellular carcinoma only	[69]
	↑		↑				↑		[70]
						↑			[71]
						↑			[72]
	↓	↑	↓	↑	↑	↑	↔		[73]
Lung cancer						↓			[46]
						↓			[74]
				↑					[75]
			↑	↑					[76]
						↔		supplementary finding: ABHD6 upregulation	[77]
						↑			[78]
Mantle cell lymphoma			↑						[79]
			↑	↑	↓		↑		[80]
Medulloblastoma			↑	↔				SHH subtype (highly aggressive medulloblastoma tumour characterised by activation of the Sonic Hedgehog (SHH) pathway originating from granule cell precursors of the developing cerebellum) with higher CB_1_ expression levels than other medulloblastoma subtypes; CB_2_ receptor expression was similar among the subtypes	[63]
Melanoma				↑					[81]
						↑			[82]
Meningioma	↓	↔							[35]
	↑	↑							[66]
			↔	↔					[39]
Mobile tongue squamous cell carcinoma			↑	↑				increased CB_2_ receptor and concomitant CB_1_ receptor/CB_2_ receptor expression was observed significantly more frequently in female than in male patients with squamous cell carcinoma of the mobile tongue	[83]
Nasopharyngeal carcinoma						↑			[84]
Ovary cancer						↑			[18]
						↓			[46]
			↑						[85]
Pancreatic cancer	↔	↔	↑	↔	↓	↓		endocannabinoid regulation compared with healthy controls; indicated cannabinoid receptor, FAAH or MAGL up- or downregulations refer to lower survival time	[86]
Pituitary adenomas	↑	↑							[87]
Prostate cancer					↑			differences in the expression of FAAH depending on the Gleason score of the tumour tissue could not be deduced	[88]
			↑						[89]
			↑						[90]
					↑				[91]
						↑			[92]
Renal cell carcinoma			↓					refers to analyses of clear renal carcinoma samples	[93]
			↓					refers to analyses of clear renal carcinoma samples	[94]
						↓			[46]
				↑					[95]
Retinoblastoma			↑						[96]
Rhabdomyosarcoma			↑						[97]
Thyroid malignancies						↔			[46]
			↑	↑					[98]
Different cancer types	↓	↑						endocannabinoids measured cross-sectionally in the plasma of age- and sex-matched subgroups of subjects that included 42 control subjects and 44 cancer patients, with no assignment to tumour types at data presentation	[99]

The arrows in the table indicate up- or downregulation in the indicated cancer tissue or serum/plasma compared to non-cancer tissue or serum/plasma. In some cases, comparisons with healthy tissue have not been performed [37,48,54,76]. Here, lower or higher expression refer to different malignancy grades. In the comments, reference is made in each case to the specific features of the studies cited. ↔, not regulated; ↑, upregulated; ↓, downregulated; 2-AG, 2-arachidonoylglycerol; ABHD6, α/β-hydrolase domain containing 6; AEA, *N*-arachidonoylethanolamine (anandamide); CB_1_, CB_2_, cannabinoid receptor 1 or 2; FAAH, fatty acid amide hydrolase; HER2, human epidermal growth factor receptor 2; MAGL, monoacylglycerol lipase; NAPE-PLD, *N*-acyl phosphatidylethanolamine phospholipase D.

**Table 2:** Relationship between expression levels of cannabinoid receptors, FAAH and MAGL and patient survival.

Tumour Type	Essential Result of the Studies	CB_1_	CB_2_	FAAH	MAGL	Reference
Breast cancer	No correlation of FAAH expression with disease-specific survival, but levels of FAAH significantly increased in patients with higher number of axillary lymph node metastases			↔		[45]
	Strong association between higher CB_2_ protein expression in HER2+ breast tumours and lower patient overall, relapse-free and metastasis-free survival		○			[47]
	High HER2–CB_2_ heteromer expression associated with lower disease-free and overall patient survival		○			[48]
Colorectal cancer	Higher CB_1_ expression correlated with poorer overall survival in stage IV; CB_1_ expression not correlated with patient survival following surgery in stage I/II or III cancer	○				[54]
	CB2 mRNA expression as prognostic factor for colon but not for rectal cancer; five-year overall survival for patients without CB2 expression was 76.16% versus 41.94% for patients with CB2 expression		○			[55]
	Higher levels of MAGL or lower levels of CB_2_ in tumour-associated macrophages of patients with colorectal cancer associated with better survival		○		○	[57]
Esophageal squamous cell carcinoma	Overexpression of CB_1_ in esophageal squamous cell carcinoma correlated with metastasis to lymph nodes and distant organs, and poor prognosis	○				[64]
Head and neck squamous cell carcinoma	Higher CB_2_ receptor expression associated with reduced disease-specific survival; CB_1_ receptor immunoreactivity not associated with survival	↔	○			[68]
Hepatocellular carcinoma	Disease-free survival in patients with hepatocellular carcinoma with high CB_1_ and CB_2_ expression significantly better than in patients with low expression	○	○			[69]
	MAGL low-expression group with significantly better survival than MAGL high-expression group				○	[71]
	Clinical prognosis for the MAGL high group markedly poorer than that for the MAGL low group in the 1-, 3-, and 5-year overall survival times and recurrence rates				○	[72]
Lung cancer	Lung adenocarcinoma patients with high CB_2_ level showed a shorter overall survival		○			[75]
	Patients with high expression levels of CB_1_, CB_2_ and CB_1_/CB_2_ showed increased survival	○	○			[76]
	Overall survival gradually reduced with increasing ABHD6 levels; no significant association with MAGL expression				↔	[77]
	High MAGL expression associated with worse outcomes				○	[78]
Mobile tongue squamous cell carcinoma	High CB_1_ and CB_2_ expression associated with longer overall and disease-free survival times	○	○			[83]
Pancreatic cancer	Correlation between longer survival and low CB_1_ receptor or high FAAH as well as MAGL levels; no correlation between survival and CB_2_ immunoreactivity	○	↔	○	○	[86]
Prostate cancer	High CB_1_ expression associated with a shorter survival time	○				[89]
	High tumour epithelial FAAH associated with a poor disease-specific survival			○		[91]
Renal cell carcinoma	Higher CB_2_ expression tending to have poor clinical outcomes in survival analyses		○			[95]

○, higher expression is associated with poorer survival (or vice versa: lower expression is associated with prolonged survival); ○, lower expression is associated with poorer survival (or vice versa: higher expression is linked with prolonged survival); ↔, no association of the indicated parameter with patients’ survival; CB_1_, CB_2_, cannabinoid receptor 1 or 2; FAAH, fatty acid amide hydrolase; HER2, human epidermal growth factor receptor 2; MAGL, monoacylglycerol lipase.
